# Curved multiplanar reformatting provides improved visualization of hippocampal anatomy

**DOI:** 10.1002/hipo.23177

**Published:** 2019-11-19

**Authors:** Donald William Gross, Ehsan Misaghi, Trevor A. Steve, Alan H. Wilman, Christian Beaulieu

**Affiliations:** ^1^ Division of Neurology, Department of Medicine University of Alberta Edmonton Alberta Canada; ^2^ Department of Biomedical Engineering University of Alberta Edmonton Alberta Canada

**Keywords:** curved multiplanar reformatting, ex vivo MRI, hippocampus

## Abstract

There is a growing body of literature studying changes in hippocampal subfields in a variety of different neurological conditions, but this work has mainly focused on the hippocampal body given challenges in visualization of hippocampal anatomy in the head and tail when sectioned in the typical coronal image plane. Curved multiplanar reformatting (CMPR) is an image reconstruction method that can improve visualization of complex three‐dimensional structures. The objective of this study was to determine whether CMPR could facilitate visualization of the human hippocampal anatomy along the entire caudal–rostral axis. CMPR was applied to high‐resolution magnetic resonance imaging acquired ex vivo on four cadaveric hippocampal specimens at 4.7 T (T2‐weighted, 0.2 × 0.2 × 0.5 mm^3^). CMPR provided clear visualization of the classic “interlocking C” appearance of the dentate gyrus and cornu ammonis along the entire caudal–rostral axis including the head and tail, which otherwise show complex anatomy on the standard coronal slices. CMPR facilitated visualization of hippocampal anatomy providing the impetus to develop simplified approaches to delineate subfields along the entire hippocampus including the usually neglected head and tail.

1

The hippocampus is a complex tubular structure made up of the cornu ammonis (hippocampus proper) and dentate gyrus with the cornu ammonis being further divided into Subregions CA1, CA2, CA3, and CA4 (Duvernoy, Cattin, & Risold, 2013). Viewed in cross section through its mid portion (the hippocampal body), the hippocampus demonstrates an “interlocking C” profile formed by the principal cell layers of the cornu ammonis and dentate gyrus, which is a remarkably consistent feature of the hippocampus for all mammals (Figure 1) (Duvernoy et al., 2013; Gloor, 1997; Golgi, 1886). The human hippocampus develops further complexity with digitations (bulges separated by shallow grooves) creating a segmented appearance along its long axis with the medial curvature of the caudal (head) and rostral (tail) ends of the structure resulting in its crescent shape (Supporting Information Video S1, Figure 2).

**Figure 1:**
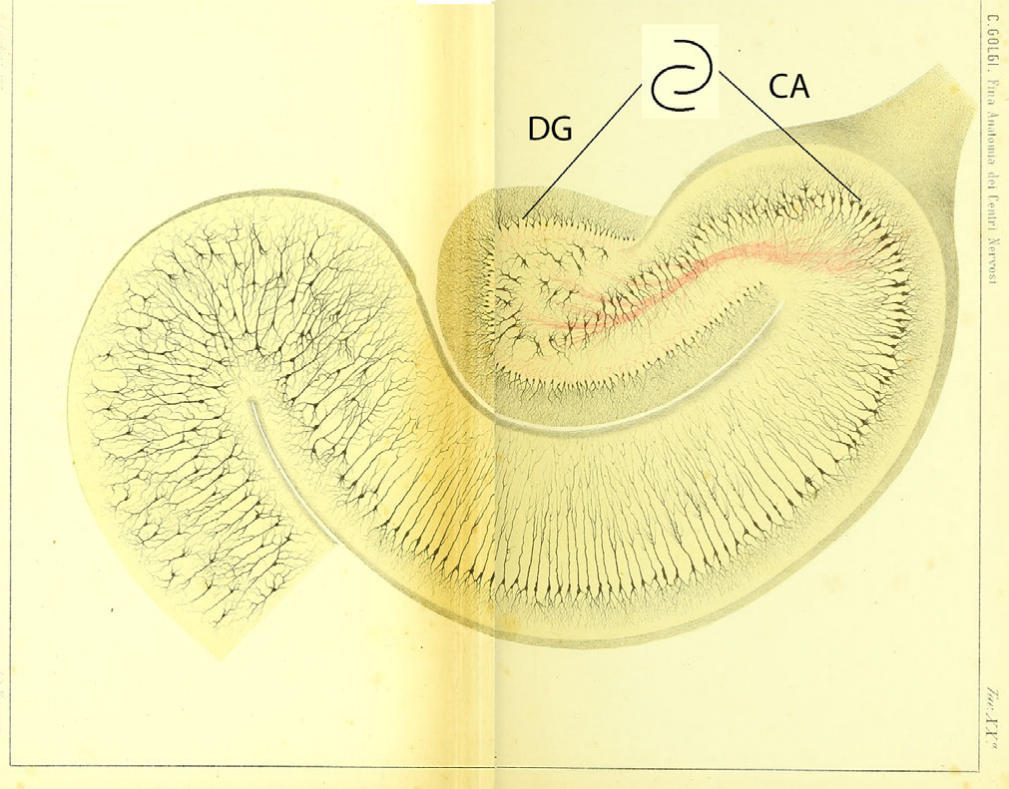
Cross‐sectional anatomy of the human hippocampus demonstrating the “interlocking C” relationship of the dentate gyrus (DG) and cornu ammonis (CA) (Golgi, 1886) (public domain) [Color figure can be viewed at http://wileyonlinelibrary.com]

**Figure 2:**
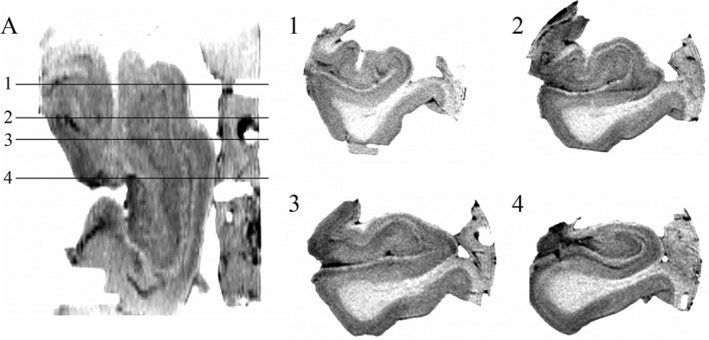
Ex vivo axial T2‐weighted image with inverted contrast of Hippocampus 1 (A) with typical “coronal” sections through the hippocampal head (Slices 1–3) and body (Slice 4). The stratum laconosum moleculare (SLM) and the “interlocking C” relationship of the dentate gyrus (DG) and hippocampus are clearly visualized in the body (Slice 4). For the head, however, while the SLM is readily visualized, the orientation of the slices in relation to the hippocampal digitations obscures the anatomical relationship between the DG and cornu ammonis (CA, Slices 1–3)

The complex three‐dimensional geometry of the human hippocampus results in significant challenges in visualization of the structure when dissected, as is typical, perpendicular to the anterior commissure–posterior commissure line. While the classical “interlocking C” relationship of the cornu ammonis and dentate gyrus is readily visualized on a coronal section through the body, the anatomical relationship between the cornu ammonis and dentate gyrus is more challenging to demonstrate in the hippocampal head and tail due to the medial curvature of the caudal and rostral portions of the hippocampus and the variable size and number of digitations at the caudal end (as shown in a high‐resolution ex vivo T2‐weighted image in Figure 2).

Histological studies have demonstrated that the hippocampal subregions can be affected specifically in different conditions, which has resulted in a rapidly expanding interest in assessing subfields in normal aging (Daugherty, Bender, Raz, & Ofen, 2016) as well as a wide range of disease states such as epilepsy, depression, Alzheimer's disease, and Parkinson's disease (Blumcke et al., 2013; de Flores, La Joie, & Chetelat, 2015; Malykhin & Coupland, 2015; Pereira et al., 2013). With advances in the spatial resolution of images acquired on modern magnetic resonance imaging (MRI) scanners, a growing literature devoted to the study of hippocampal subfields in vivo has developed (Yushkevich et al., 2015). Hippocampal subfield segmentation is based on distinct histological differences which define the subfield boundaries (Ding & Van Hoesen, 2015; Duvernoy et al., 2013). As these histological features are not visible on MRI, segmentation protocols have been developed based on geometric rules derived from anatomical specimens with recent studies providing histological validation of some segmentation protocols (Steve et al., 2017). The majority of hippocampal segmentation protocols have been developed for the hippocampal body with the segmentation of the hippocampal head having been demonstrated to show the greatest disagreement among protocols (Yushkevich et al., 2015). The MRI demonstration of variability of hippocampal changes along the caudal–rostral axis with aging (Malykhin, Bouchard, Camicioli, & Coupland, 2008) and in disease states such as Parkinson's disease and epilepsy (Bouchard et al., 2008; Thom et al., 2012) emphasize the importance of developing methods to study hippocampal subfields along the entire length of the structure.

While the complex three‐dimensional structure of the human hippocampus presents challenges to visualize, as pointed out by Gloor: “The most important visual concept to keep in mind is that the two interlocking half cylinders formed by the hippocampus proper (cornu ammonis) and the dentate gyrus, which in cross sections through the body form the classical double‐C profile of the hippocampus and dentate gyrus, retain their relationship with each other regardless of the various curvatures the hippocampus undergoes along its caudorostral extent” (Gloor, 1997). In order to illustrate this concept, the coxcomb oyster fossil provides a useful visual aid given the structural similarities between the fossil and the human hippocampus (Figure 3). As with the hippocampus, the coxcomb oyster is a tubular structure with the ridges of the oyster shell being comparable to the hippocampal digitations. Both the coxcomb oyster and the hippocampus are curved into a crescent shape with the segmented structure maintaining its orientation to the arc of the crescent as opposed to respecting the anterior–posterior axis (Figure 3).

**Figure 3:**
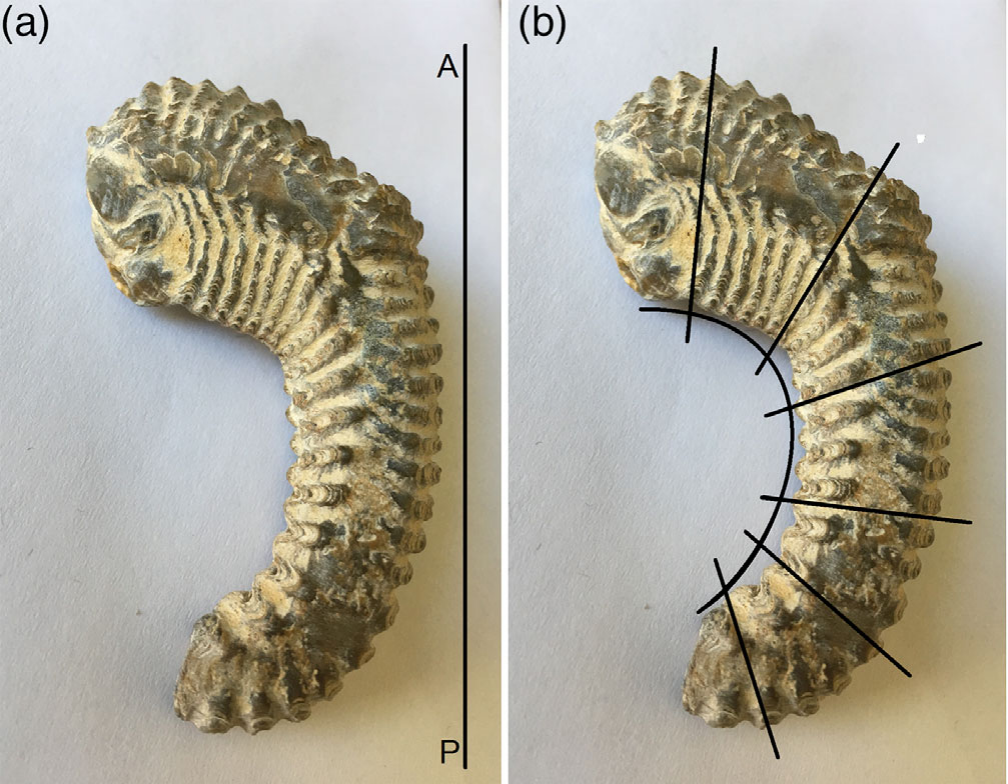
The coxcomb oyster fossil demonstrates striking structural similarity to the human hippocampus. (a) The oyster fossil arcs such that at the anterior and posterior ends the ridges of the shell do not respect that anterior–posterior axis (A–P). (b) The ridges of the fossil shell are oriented orthogonal to the arc of the fossil [Color figure can be viewed at http://wileyonlinelibrary.com]

Three‐dimensional curved multiplanar reformatting (CMPR) has been used to improve visualization of tubular structures with complex three‐dimensional orientation such as blood vessels and the tracheobronchial tree (Remy, Remy‐Jardin, Artaud, & Fribourg, 1998). By choosing planes of view that continually change orientation in order to remain transverse to the long axis of the curved structure of interest, CMPR can facilitate the depiction of pathology, which may not be apparent on conventional rectilinear planes (Remy et al., 1998). CMPR has recently been applied to the hippocampal tail on ex vivo human specimens scanned at 9.4 T demonstrating “body‐like appearance” (i.e., “interlocking C”) of the posterior hippocampus on resliced sections (Adler et al., 2018). Based on the expectation that the classical “interlocking C” relationship of the cornu ammonis (hippocampus proper) and dentate gyrus is preserved throughout the entire hippocampus including the head, CMPR was applied here to ex vivo MRI to facilitate the visualization of hippocampal anatomy in the head and tail.

Ethics approval was obtained from the institutional research ethics board.

Four hippocampi from different individuals with no history of neurological disease were obtained postmortem (Hippocampus 1–75 year old male, Hippocampus 2–61 year old male, Hippocampus 3–91 year old female, Hippocampus 4–87 year old male). Specimens were fixed in formaldehyde for an average of 6 months, trimmed to fit into a 50 ml centrifuge tube and immersed in a liquid fluorocarbon (Fluorinert, 3M). Specimens were scanned at room temperature in a 4.7 T MRI (Unity Inova, Varian, Palo Alto, CA) using a custom‐built volume radiofrequency (RF) coil (38 mm inner diameter, 38 mm long) and a T2‐weighted (inverted contrast) fast‐spin echo technique with 80 contiguous 0.5 mm coronal slices perpendicular to the long axis of the body of the hippocampus (TE = 39 ms, TR = 10,000 ms, FOV = 40 × 40 mm^2^, in plane matrix = 200 × 200, one average, native resolution of 0.2 × 0.2 × 0.5 = 0.02 mm^3^, scan time 9 min). As expected, there was marked signal attenuation at the ends of the short length RF coil. While it was possible to scan Hippocampus 1 completely, only the hippocampal head and body were successfully imaged for the remaining three hippocampi.

CMPR was performed using 3D Slicer version 4.8.1 (https://www.slicer.org). The selection of the plane of view was performed manually in order to maintain a transverse orientation to the long axis of the curved hippocampus (Figure 4). To achieve this objective, the orientation of the plane of view was adjusted obliquely in the caudal and rostral ends of the hippocampus using the crests and troughs of the digitations as landmarks to assist in maintaining a tangential orientation to the curved arc of the hippocampus. Based on the hypothesis that consistent cross‐sectional anatomy would be observed along the entire length of the hippocampus, the plane of visualization was manually adjusted in order to achieve the orientation that best demonstrated the “interlocking C” relationship between the cornu ammonis and the dentate gyrus for each slice. For Hippocampus 1, CMPR was performed at 1 mm intervals along the entire hippocampus providing a total of 93 slices (Supporting Information Video S1). Due to the above described signal attenuation in the hippocampal tail, CMPR was only performed at 1 mm intervals for the hippocampal head and body for Hippocampi 2–4.

**Figure 4:**
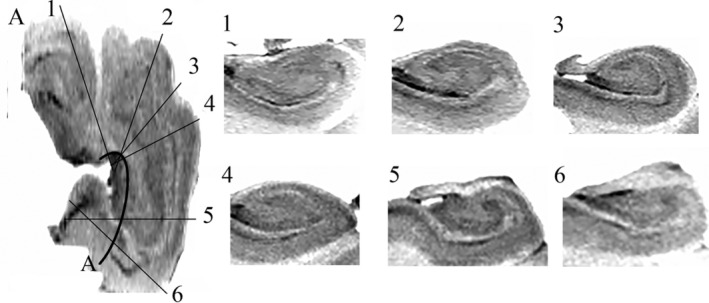
(A) Ex vivo axial T2‐weighted image with inverted contrast of Hippocampus 1. Slices tangential to the arc of the hippocampus (A) were obtained using curved multiplanar reformatting (CMPR) (Slices 1–6). Sections through the head (Slices 1–4), body (Slice 5), and tail (Slice 6) all demonstrate the “interlocking C” relationship of the dentate gyrus (DG) and cornu ammonis (CA) that were not observed on the head coronal slices in Figure 2

The feasibility of applying a single common method of hippocampal subfield segmentation to the hippocampal head, body, and tail was assessed for Hippocampus 1. Subfield segmentation was performed on slices obtained with CMPR for the head, body, and tail based on our previously published manual protocol providing the following subfields: CA1, CA2, and CA3/CA4/dentate gyrus (Figure 6) (Steve et al., 2017).

The internal architecture of the hippocampus, including the stratum lacunosum moleculare (SLM), dentate gyrus, and cornu ammonis is clearly visualized with ex vivo imaging (Figure 2). Hippocampal digitations are observed which are most prominent in the hippocampal head with variability in the number and depth of digitations as well the angle of the medial curvature of the head of the hippocampus observed between specimens (Figures 2 and 5). When viewing the hippocampus in the coronal plane (Figure 2), the “interlocking C” relationship of the dentate gyrus and cornu ammonis is clearly visualized in the body. For the head, however, while the SLM is readily visualized, the orientation of the slices in relation to the hippocampal digitations results in the SLM, dentate gyrus and cornu ammonis having a complex, irregular morphology where the anatomical relationship between the dentate gyrus and cornu ammonis is difficult to determine (Figures 2 and 5). Applying CMPR to Hippocampus 1, the “interlocking C” relationship of the dentate gyrus and cornu ammonis can be seen throughout the entire caudal–rostral extent of the hippocampus (Figure 4, Animation 1). Further CMPR performed on the head of Hippocampi 2–4 also demonstrates clear visualization of the “interlocking C” relationship of the dentate gyrus and cornu ammonis which is not obvious on a standard coronal slice orientation (Figure 5). The demonstration of a consistent anatomical relationship preserved along the entire length of the hippocampus, permits the application of a single segmentation protocol to the entire hippocampus as demonstrated in Figure 6.

**Figure 5:**
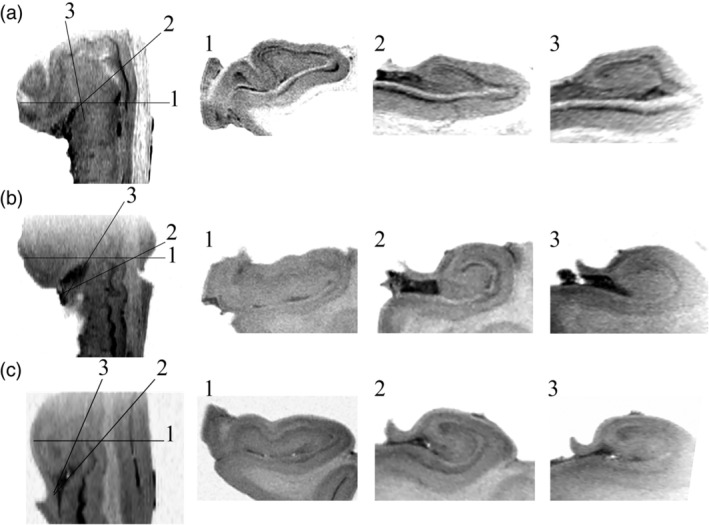
Ex vivo axial T2‐weighted images with inverted contrast of hippocampal head and body for Hippocampus 2 (a), Hippocampus 3 (b), and Hippocampus 4 (c). Standard coronal sections through the head of all three hippocampi demonstrates a complex relationship between the dentate gyrus (DG) and cornu ammonis (CA) (1) while curved multiplanar reformatting (CMPR) demonstrates the “interlocking C” relationship is preserved in the hippocampal head (2, 3)

**Figure 6:**
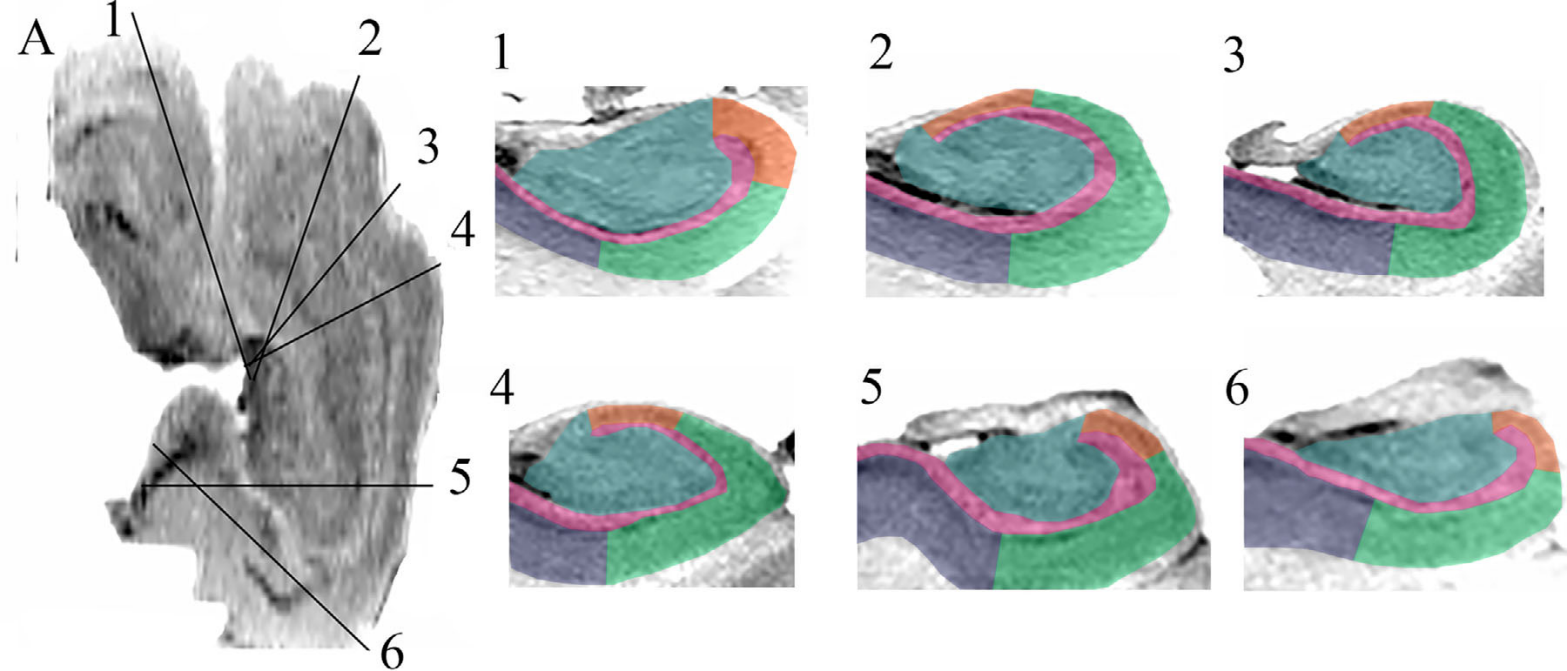
Hippocampal subfield labels are demonstrated on curved multiplanar reformatting (CMPR) sections along the entire hippocampal axis (pink, SLM; mauve, subiculum; green, CA1; orange, CA2; blue, CA3/CA4/dentate gyrus [DG]) [Color figure can be viewed at http://wileyonlinelibrary.com]

While tremendous advances have been made regarding the segmentation of the hippocampal body (Wisse et al., 2017; Yushkevich et al., 2015), segmentation of the hippocampal head and tail have been more challenging based on the more complex relationship of the dentate gyrus and cornu ammonis as visualized in the typically used coronal plane. A detailed cytoarchitecture and chemoarchitecture‐based parcellation of the hippocampal head and body has provided critical histological reference atlases based on two, three and four digitation models (Ding & Van Hoesen, 2015). This has led to novel approaches for the segmentation of the hippocampal head (Berron et al., 2017; Dalton, Zeidman, Barry, Williams, & Maguire, 2017); however, limitations apply to both the atlases and segmentation methods. Specifically, the atlases do not account for lesser and greater number of hippocampal digitations which can occur. As well, the angle of curvature of the hippocampus at the head and tail can vary dramatically which could also impact reliability (DeKraker, Ferko, Lau, Kohler, & Khan, 2018). A novel method of hippocampal segmentation based on unfolding of the hippocampus into two‐dimensional space around the manually traced SLM has been reported (DeKraker et al., 2018). This method provided encouraging results, in particular given the independence on the number hippocampal digitations and the ability to perform well along the entire length of the hippocampus (DeKraker et al., 2018). A limitation of this method, however, is that clear visualization of the familiar anatomy tangential to the axis of the hippocampus is not obtained.

CMPR application to proton density‐weighted images of the hippocampal tail in ex vivo human samples imaged at 9.4 T has demonstrated consistent “body‐like” appearance (i.e., “interlocking C”) of resliced sections of the posterior hippocampus suggesting that CMPR can facilitate visualization of the hippocampal tail (Adler et al., 2018). CMPR was not however applied to the caudal (head) hippocampus. The application here of CMPR to the entire hippocampus (Hippocampus 1) and the hippocampal head in three additional cases (Hippocampi 2–4), provides confirmation of Gloor's anatomical observations of the dentate gyrus and cornu ammonis as two interlocked half cylinders which is preserved along the entire length of the hippocampus including the head, body and tail (Animation 1) (Gloor, 1997). Along with the application to ex vivo MRI, CMPR has the potential to be applied to in vivo MRI as well as histopathology (which is also typically sectioned perpendicular to the hippocampal body) in order to facilitate visualization of hippocampal anatomy along the entire long axis of the structure. Further, the demonstration of a consistent relationship of the cornu ammonis and dentate gyrus throughout the entire hippocampus would suggest that complex segmentation protocols designed to account for heterogeneity of digitations and curvature may not be necessary but rather that it should be possible to obtain reliable segmentation of the entire hippocampus using a single approach as demonstrated in Hippocampus 1 (Figure 6).

While we have successfully applied the CMPR method of visualizing hippocampal internal architecture on very high resolution T2‐weighted MRI in four ex vivo hippocampal specimens, a limitation of this technique is that the CMPR approach was manual, making it susceptible to user bias and being labor intensive. The sharp anterior curve of the arc of the hippocampus anteriorly, could result in variability in the selection of the orientation of the imaging plane between users and it will therefore be necessary to determine inter and intra rater reliability in particular if CMPR is used to measure hippocampal subfield volumes. While the application of our previously reported methods for segmenting the hippocampal body to the CMPR data demonstrates feasibility, we were unable to confirm the accuracy of the labeling as our specimens were sectioned orthogonal to the A‐P axis. Despite this limitation, our preliminary results are encouraging and suggest that CMPR could be applied to ex vivo MRI, histology, and potentially in vivo MRI in order to facilitate visualization and measurement of hippocampal subfields along the entire length of the hippocampal axis.

## CONFLICT OF INTEREST

The authors declare that they have no conflict of interest.

2

## Supporting information


**Video S1** Curved multiplanar reformatting demonstrates that the “interlocking C” relationship between the cornu ammonis and dentate gyrus is maintained throughout the entire length of Hippocampus 1.

## Data Availability

The data that support the findings of this study are available from the corresponding author upon reasonable request.
